# Mitochondrial Cation Signalling in the Control of Inflammatory Processes

**DOI:** 10.3390/ijms242316724

**Published:** 2023-11-24

**Authors:** Pampa Pain, Francesca Spinelli, Gaia Gherardi

**Affiliations:** Department of Biomedical Sciences, University of Padova, 35131 Padova, Italy; pampa.pain@phd.unipd.it (P.P.); francesca.spinelli.1@studenti.unipd.it (F.S.)

**Keywords:** mitochondrial Ca^2+^ uptake, inflammation, mitochondrial K^+^ flux

## Abstract

Mitochondria are the bioenergetic organelles responsible for the maintenance of cellular homeostasis and have also been found to be associated with inflammation. They are necessary to induce and maintain innate and adaptive immune cell responses, acting as signalling platforms and mediators in effector responses. These organelles are also known to play a pivotal role in cation homeostasis as well, which regulates the inflammatory responses through the modulation of these cation channels. In particular, this review focuses on mitochondrial Ca^2+^ and K^+^ fluxes in the regulation of inflammatory response. Nevertheless, this review aims to understand the interplay of these inflammation inducers and pathophysiological conditions. In detail, we discuss some examples of chronic inflammation such as lung, bowel, and metabolic inflammatory diseases caused by a persistent activation of the innate immune response due to a dysregulation of mitochondrial cation homeostasis.

## 1. Introduction

Mitochondria are dynamic organelles present in all eukaryotic cells essential to sustain cellular energy balance.

They are formed by double membranes; the outer mitochondrial membrane (OMM) is freely permeable to ions and metabolites up to 5 kDa, while the inner mitochondrial membrane (IMM) is impermeable to most ions and small molecules due to a tight diffusion barrier, allowing only tiny molecules and ions to cross into the matrix with the help of selective channels and transporters. The IMM is characterized by archetypal invaginations called cristae, which enclose the matrix defining the internal compartments, the cristae junctions, that limit the diffusion of molecules from the intra-cristae space towards the intermembrane space (IMS), thus creating a micro-environment wherein the mitochondrial Electron Transport Chain (ETC) complexes are hosted.

Mitochondria are characterized by a negative electrochemical proton potential of −180 mV which is necessary to sustain the ATP synthase for ATP production in the oxidative phosphorylation process [1]. Mitochondria play a key role in the regulation of cell survival and death, since they are involved in energy metabolism as well as in the regulation of both necrotic and apoptotic cell death pathways. In addition, they play fundamental roles in many physiological aspects, such as the synthesis of several amino acids, lipid metabolism, and ROS production. All these processes are mainly sustained by ion homeostasis; thus, the maintenance of the internal membrane integrity is essential to regulate survival and cell death. The IMM is equipped with several ion transporters and ion channels to maintain ion homeostasis. In particular, Ca^2+^ and K^+^ are the two most crucial ions that regulate mitochondrial functions and swelling [2].

Moreover, recent findings indicate that mitochondria are necessary for the induction and maintenance of innate and adaptive immune systems, in particular, in inflammatory events [3]. In general, inflammation is driven by the activation of pattern recognition receptors (PRRs). However, PRRs can be activated either by bacterial or viral infections, known as pathogen-associated molecular patterns (PAMPs), or by endogenous molecules, which are commonly called “damage-associated molecular patterns” (DAMPs) [4]. Mitochondrial DAMPs (mtDAMPs) are molecules that are released from mitochondria to extracellular space during cell death and stress responses; they include not only proteins but also DNA or lipids, like mitochondrial DNA (mtDNA), adenosine triphosphate (ATP), succinate, and cardiolipin. The release of mtDAMPs induces inflammatory responses and can be associated with the development of chronic inflammatory diseases [5]. In this context, mitochondria, by participating in ion homeostasis (Ca^2+^ and K^+^), act as signalling platforms and mediators in effector responses. In detail, the dysregulation of mitochondrial Ca^2+^ and K^+^ homeostasis can induce oxidative stresses, apoptotic and necrotic events due to Ca^2+^ overload and dysregulation of the matrix volume, and all these events seem to cause mtDAMP release, regulating inflammation [6,7].

In this review, we will summarize how mitochondria crosstalk and regulate the immune system. In detail, we aim to unveil the molecular signalling triggered by mitochondrial Ca^2+^ accumulation and mitochondrial K^+^ flux in the inflammatory response. Finally, we will discuss some examples of chronic inflammatory diseases caused by persistent activation of the immune response, as well as cases of neurodegenerative diseases caused by a dysregulation of mitochondrial cation homeostasis.

## 2. Mitochondrial Cation Homeostasis

### 2.1. Mitochondrial Ca^2+^ Signalling

Ca^2+^ is a critical second signal that regulates many physiological and pathological functions. Indeed, it modulates cellular proliferation, differentiation, metabolism, muscle contraction, synaptic plasticity, and cell death. Consequently, Ca^2+^ homeostasis must be highly controlled [8].

The [Ca^2+^] in the extracellular environment is maintained at around 1–2 mM, whereas in the cytosol, it is maintained at a low concentration of 100 nM in resting conditions but is able to increase to up to 1–3 µM when the system is stimulated [9]. The rapid increase in [Ca^2+^]_cyt_ is one of the main features of Ca^2+^ signalling at the cellular level. Ca^2+^ is mobilized from different external sources or internal stores. We can distinguish three categories of Ca^2+^ channels in the plasma membrane: Voltage-Operated Ca^+2^ Channels (VOCCs), Receptor-Operated Ca^2+^ Channels (ROCCs), and Store-Operated Ca^+2^ Channels (SOCCs); they mediate Ca^2+^ entry from the extracellular environment upon stimulation [10]. In general, store-operated Ca^2+^ entry (SOCE) serves to replenish ER and modulate cytosolic Ca^2+^ signalling, thereby regulating a variety of cellular functions. Mitochondria play a crucial role in shaping cytosolic Ca^2+^ waves, thanks to the presence of the MCU complex and their proximity to Calcium Release-Activated Ca^2+^ (CRAC) channels. This strategic localization ensures that CRAC channels are shielded from Ca^2+^-dependent inactivation, a process they are susceptible to once activated. Thus, the mitochondrial control of cytosolic Ca^2+^ waves emerges as a pivotal mechanism in preserving the functionality of SOCE [11]. Moreover, Ca^2+^ can be released in the cytosol from internal stores: the endoplasmatic and sarcoplasmatic reticuli (ER/SR) and Golgi. In ER and SR, [Ca^2+^] levels are among 400–500 µM. Ca^2+^ is released from the ER through inositole-1,4,5-triphosphate receptors (InsP_3_R), activated by different external stimuli leading to the formation of IP_3_ [12].

Finally, Ca^2+^ is removed from the cytoplasm through the Na^+^/Ca^2+^ exchanger (NCX), characterized by low affinity, and through pumps with ATPase activity, such as the sarco-endoplasmic reticulum ATPases (SERCAs), which allow the entry of Ca^2+^ into the ER, and plasma membrane Ca^2+^-ATPase pumps (PMCAs), which pump Ca^2+^ back into the extracellular space [13].

**Figure 1:**
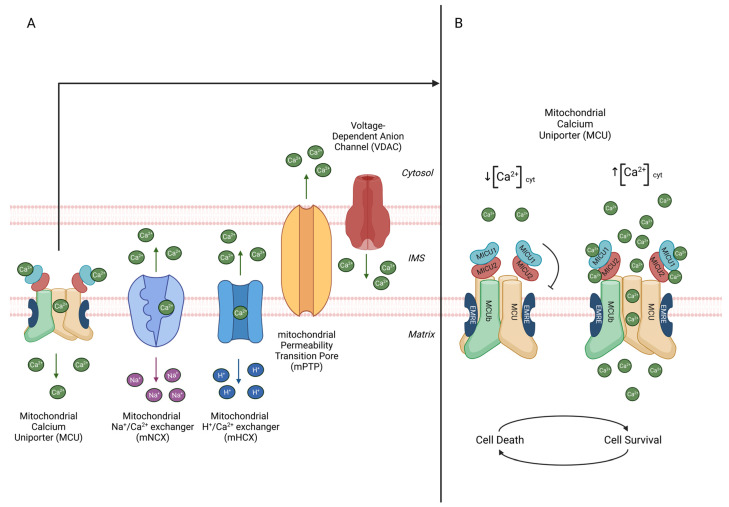
(**A**) Ca^2+^ freely crosses the OMM thanks to the presence of the voltage-dependent ion channels (VDACs); therefore, it enters the mitochondrial matrix through the mitochondrial calcium uniporter (MCU). Nevertheless, the exit of Ca^2+^ from the matrix is mediated by Na^+^/Ca^2+^ antiporter (NCLX) and H^+^/Ca^2+^ exchanger (HCX). Finally, a mitochondrial Ca^2+^ overload can induce apoptosis by the opening of the mitochondrial permeability transition pore (mPTP). (**B**) MCU is a highly selective Ca^2+^ channel. It is a complex that comprises pore-forming subunits (MCU, MCUb, and EMRE) inserted in the IMM and regulatory subunits (MICU1, MICU2) facing the intermembrane space. At high cytosolic Ca^2+^ concentrations, the MICU1–MICU2 heterodimer binds calcium ions and acts as positive regulator of MCU activity, allowing mitochondrial calcium uptake. Ca^2+^ in the mitochondrial matrix acts as second signal, mediating both cell survival and cell death.

Mitochondria are essential modulators of Ca^2+^ signalling since they store high [Ca^2+^] in the matrix due to the negative mitochondrial membrane potential (MMP). [Ca^2+^] in the mitochondrial matrix ([Ca^2+^]_mit_) is extremely low in resting conditions, comparable to that in the cytosolic one. Nevertheless, it rapidly increases in response to a physiological stimulus. The latter causes a transient [Ca^2+^]_cyt_ increase from 0.1 μM to 2–3 μM and a parallel rise in [Ca^2+^]_mit_ [9]. Ca^2+^ ions freely cross the OMM thanks to the presence of voltage-dependent ion channels (VDAC) that allow the entry of ions and small molecules (MW < 10 kDa) [14]. On the contrary, the IMM is ion-impermeable. Therefore, Ca^2+^ enters the mitochondrial matrix through a highly selective channel, the mitochondrial Ca^2+^ uniporter (MCU) [15,16]. Finally, the exit of Ca^2+^ from the matrix is mediated by the Na^+^/Ca^2+^ antiporter (NCLX) and H^+^/Ca^2+^ exchanger that exploit the electrochemical gradient generated by the electron transport chain [17,18] (Figure 1A).

Nevertheless, the [Ca^2+^]_cyt_ achieved upon physiological stimulation does not reach the affinity values of the MCU for Ca^2+^. However, mitochondrial Ca^2+^ channels are located close to the Ca^2+^ release sites in the ER, forming quasi-synaptic junctions called microdomains. In these regions, the local [Ca^2+^] is higher than that in the cytoplasm and sufficient to activate the opening of the MCU. This evidence attenuates the discrepancy between the low affinity of the MCU to Ca^2+^ and the amplitude of [Ca^2+^]_mit_ [19].

When [Ca^2+^]_cyt_ increases, it rapidly internalizes inside the mitochondria, which buffer variations in the intracellular environment to maintain Ca^2+^ homeostasis and sustain many different processes. Under physiological conditions, mitochondrial Ca^2+^ uptake controls the energetic cellular status by regulating the tricarboxylic acid cycle (TCA) and the ATP production [20]. Indeed, Ca^2+^ regulates three enzymes of oxidative metabolism: α-ketoglutarate and isocitrate dehydrogenase, through allosteric regulation, and pyruvate dehydrogenase, by controlling its phosphorylation state [9].

In pathological conditions, excessive mitochondrial Ca^2+^ entry leads to cell death and increases ROS production. Finally, mitochondrial Ca^2+^ overload is able to induce apoptosis by the opening of the mitochondrial permeability transition pore (mPTP), a protein complex localized in the IMM [21]. It is noteworthy that the total concentration of Ca^2+^ within mitochondria can significantly rise. However, as previously mentioned, the bioavailable free Ca^2+^ within these organelles is meticulously regulated within the micromolar range, facilitated by the interplay of two systems: Ca^2+^ exchange across the IMM and Ca^2+^ buffering in the matrix, primarily involving inorganic phosphate. Specifically, interactions between orthophosphate and Ca^2+^ play a predominant role in this buffering mechanism. Nevertheless, it is important to note that this interaction alone cannot fully account for all the Ca^2+^ buffering properties. Therefore, other forms of phosphate must be considered [22,23,24].

The mPTP is a channel characterized by its high permeability of molecules up to 1500 Daltons, responsible for membrane potential dissipation, leading to membrane depolarization, mROS generation, and the release of proapoptotic factors [21]. In addition, it is worth mentioning that membrane depolarization and increased mitochondrial ROS production can induce the opening of mPTP [25].

Given these crucial roles exerted by mitochondrial Ca^2+^, its concentration must be tightly regulated.

#### 2.1.1. Mitochondrial Ca^2+^ Uniporter Complex

The mitochondrial Ca^2+^ uniporter complex (MCUc) is a protein complex located in the IMM that mediates the Ca^2+^ influx in the mitochondrial matrix [26]. It comprises three pore-forming subunits (MCU, MCUb, and EMRE) and three regulatory subunits facing the IMS (MICU1, MCIU2, and MICU3) (Figure 1B).

MCU is a 40 kDa protein that forms the pore of the MCU [15,16]. From the structural point of view, it is composed of two coil-coiled domains and two transmembrane domains linked by a highly conserved loop. This loop is enriched in acidic residues (DIME motif) that confer the selectivity for Ca^2+^ [16,27]. The most critical residues in Ca^2+^ selectivity are E256, D260, and E236. Indeed, their substitution reduces Ca^2+^ permeability [16]. To form a functional channel, MCU oligomerizes in a tetrameric structural pore of approximately 170 kDa with a non-obvious symmetry: the transmembrane domain displays a fourfold symmetry. In contrast, the N-terminal domain facing the matrix shows a twofold symmetry axis [28]. MCU interacts with the pore-forming subunits (MCUb and EMRE), forming a tetramer to modulate the channel activity. Although MCU is sufficient to form a selective Ca^2+^ channel, the assembly of a functional complex requires the presence of EMRE, and the regulation of its opening properties involves interactions with the regulatory subunits MICU1 and MICU2 [29].

MCU genetic manipulation in different animal models highlights the critical role of mitochondrial Ca^2+^ uptake in energy production. In *Mus musculus*, the study of MCU deletion is biased by the fact that its phenotype strictly depends on the mouse strain. The first MCU knockout mouse (MCU^−/−^) generated in a mixed background (C57/BL6 and CD1) showed a relatively mild phenotype, characterized by increased plasma lactate levels upon starvation and impaired exercise performance accompanied by a reduction of skeletal muscle pyruvate dehydrogenase activity [30]. However, in a pure C57BL/6 background, MCU deletion is embryonically lethal [31]. For a comprehensive review, see [32].

MCUb is a 40 kDa protein that shares 50% sequence homology with the MCU. Similar to the MCU, it comprises two coiled-coil domains and two transmembrane domains, separated by a loop [33].

It is highly conserved in vertebrates but absent in other organisms in which MCU is present (e.g., plants). It has been demonstrated that MCUb forms oligomers with MCU. Compared to MCU, the MCUb loop presents two crucial amino acid substitutions (the murine Arg251 and Glu256 residues are substituted into Trp and Val, respectively) that drastically reduce channel conductivity. Indeed, MCUb overexpression strongly decreases mitochondrial Ca^2+^ uptake, while its silencing increases [Ca^2+^]_mit_, thus confirming its inhibitory role on channel activity. The ratio of MCU/MCUb can vary between different tissues, being very high (40:1) in skeletal muscle and lower (1:3) in the heart [34]. Interestingly, the ratio correlates with the levels of mitochondrial Ca^2+^ accumulation in these tissues. Therefore, it was hypothesized that the modulation of this ratio in different tissues could set the overall mitochondrial Ca^2+^ uptake capacity to sustain tissue-specific functions.

EMRE is a 10 kDa protein located in the IMM. It is composed of a transmembrane domain, a short N-terminal domain, and a conserved C-domain enriched in acidic residues. The EMRE N-terminus is located in the mitochondrial matrix, interacting with MCU. On the other hand, its acidic C-terminal domain faces the mitochondrial matrix and interacts with the regulatory subunits MICU1 and MICU2 [35]. As a consequence, EMRE keeps the regulatory subunits attached to MCU.

In yeast cells lacking mitochondrial Ca^2+^ uptake mechanisms, the expression of human EMRE is essential to assemble a functional channel with MCU [36]. However, in a planar lipid bilayer, MCU and the regulatory subunits MICU1 and MICU2 display channel activity without the presence of EMRE [37].

To reconcile these discrepancies, an EMRE^−/−^ mouse model was generated [38]. As for MCU^−/−^ animals, EMRE^−/−^ mice are viable only in a mixed genetic background, although they are smaller and born less frequently. No differences were detected in the basal metabolic functions, running capacity, and cardiac functionality in EMRE^−/−^ animals compared to those of the controls. Thus, similarly to the MCU^−/−^ mouse model [30,31], it was hypothesized that adaptations occur to sustain the mitochondrial activity without a functional MCU channel.

A critical aspect of mitochondrial Ca^2+^ uptake is the sigmoidal response to cytosolic Ca^2+^ levels. At low [Ca^2+^]_cyt_, mitochondrial Ca^2+^ accumulation is insignificant, despite the huge driving force caused by the MMP. When the [Ca^2+^]_cyt_ rises upon cell stimulation, the speed and amplitude of mitochondrial Ca^2+^ uptake are exponentially enhanced. This feature protects the organelle from a vain energy expenditure due to cations’ vicious cycle across the IMM and Ca^2+^ overload in resting conditions. However, the MCU protein lacks Ca^2+^-binding domains. Thus, the presence of regulatory proteins able to sense changes in [Ca^2+^]_cyt_ was postulated. These proteins have been demonstrated to be MICU1, MICU1.1, MICU2, and MICU3, which contain EF-hand Ca^2+^-binding domains, each of them characterized by different tissue expression profiles and peculiar functions.

MICU1 is the critical modulator of mitochondrial Ca^2+^ uptake [39]. It forms dimers with MICU2 to regulate the MCU activity in response to [Ca^2+^]_cyt_ variations [37]. MICU1 potentiates MCU allosterically through EMRE interaction [40], while MICU1^−/−^ reduces the channel open probability [41].

As mentioned above, MICU1 interacts with MICU2, forming heterodimers stabilized by two disulphide bonds between conserved cysteines (MICU1^C465^ and MICU2^C410^), and modulates the activity of the MCU complex. MICU2 stability depends on the presence of MICU1, as MICU1 knockdown induces MICU2 degradation. On the contrary, MICU2 silencing promotes the formation of MICU1–MICU1 homodimers [37]. In detail, in resting conditions, the inhibitory function of MICU2, the genuine gatekeeper of the channel, prevails. At low [Ca^2+^]_cyt_ (<1 μM), the MCU pore is blocked by MICU1 to prevent the uncontrolled accumulation of Ca^2+^ in the mitochondrial matrix. When the [Ca^2+^]_cyt_ increases, the inhibitory function of MICU2 is suppressed, and simultaneously, the positive activator function of MICU1 is enhanced, leading to cooperative activation of the channel. MICU1 moves away from the MCU surface upon a conformational change, thus allowing the pore to open [42]. This behaviour is one key feature of mitochondrial Ca^2+^ uptake, called the sigmoidal response.

In addition, MICU1.1, a MICU1 splice variant, is highly expressed in skeletal muscle [43]. Compared to MICU1, MICU1.1 contains an extra micro-exon between exons 5 and 6 that encodes four amino acids (EFWQ). MICU1.1 forms dimers with MICU2 and plays a gatekeeping function similar to that of MICU1. However, MICU1.1 binds Ca^2+^ one order of magnitude more efficiently than MICU1 and activates MCU at a lower [Ca^2+^] uptake than MICU1 [43]. Thus, in skeletal muscle, the MICU1.1–MICU2 dimer enhances the ability of mitochondria to accumulate Ca^2+^ and increases ATP production. Eventually, MICU3, predominantly expressed in the brain, forms disulfide bonds with MICU1, acting as a channel activator [44]. The MICU1–MICU3 dimer decreases the MCU opening threshold, ensuring high mitochondrial Ca^2+^ uptake even at low [Ca^2+^]_cyt_ [45]. MICU3 is involved in the metabolic flexibility of nerve terminals. Presynaptic mitochondria can sustain axonal ATP synthesis taking up Ca^2+^ in response to small cytosolic Ca^2+^ peaks, thanks to the fine-tuning of Ca^2+^ sensitivity exerted by MICU3 [46].

#### 2.1.2. Mitochondrial Ca^2+^ Efflux

Two major systems have been postulated to extrude Ca^2+^ from the mitochondrial matrix in response to physiological mitochondrial Ca^2+^ accumulation: the mitochondrial Na^+^/Ca^2+^ exchanger (mNCX) and the mitochondrial H^+^/Ca^2+^ exchanger (mHCX). The mNCX is the predominant antiporter in excitable tissues, whereas the mHCX is particularly active in non-excitable tissues. The stoichiometry of the mNCX is defined as electrogenic, with three (or four) Na^+^ exchanged in exchange for one Ca^2+^ [47], whereas the exchange ratio of the mHCX is electroneutral (two H^+^ for one Ca^2+^) [48]. Recently, mNCX function was ascribed to NCLX [17]. NCLX mediates not only Na^+^/Ca^2+^ exchange but also Li^+^-dependent Ca^2+^ transport. Electron microscopy and cell fractionation experiments showed that NCLX is located in the IMM. Na^+^-dependent Ca^2+^ release was strongly reduced in NCLX-knockdown cells, whereas it was enhanced upon NCLX overexpression [17]. The idea that NCLX encodes the mNCX is corroborated by the discovery that NCLX-driven Ca^2+^ extrusion is inhibited by 7-Chloro-5-(2-chlorophenyl)-1,5-dihydro-4,1-benzothiazepin-2 (3H)-one (CGP-37157), the most selective inhibitor of mitochondrial Na^+^/Ca^2+^ exchange [17].

On the other hand, the mitochondrial H^+^/Ca^2+^ exchanger (CHX) was discovered in the 1970s by Carafoli and co-workers, who underlined its role in maintaining Ca^2+^ homeostasis [18]. Later, the LETM1 protein was proposed as the CHX [49], and it seems to be involved in Ca^2+^/K^+^ transport [50]. Finally, defining the mitochondrial interactome of LETM1, TMBIM5 (Transmembrane BAX inhibitor-1 Motif 5) was discovered [51]. It is the only TMBIM family member with mitochondrial localization and has been identified as a CHX. It physically interacts with LETM1 to maintain the Ca^2+^ balance. Moreover, it has been demonstrated to reduce Na^+^-independent mitochondrial Ca^2+^ release in TMBIM5^−/−^ mutants [51]. This evidence was confirmed by another work, which identified TMBIM5 as a CHX in the IMM [52]. Moreover, it has been demonstrated to bind and inhibit the m-AAA protease, ensuring cell survival, allowing mitochondrial Ca^2+^ efflux, and limiting mitochondrial hyperpolarization [52]. However, a further study investigated the role of TMBIM5 in the mitochondrial ion homeostasis field, concluding that TMBIM5 is a novel component of the mitochondrial ion transport machinery affecting Ca^2+^ and K^+^ ions [53]. The loss of TMBIM5 is correlated with increased K^+^ concentration and reduced H^+^ levels in the mitochondrial matrix [53]. In conclusion, three independent works studied the role of the TMBIM5 protein; Nowikosky and Patron demonstrated that TMBIM5 is the putative mitochondrial CHX, whereas Zhang did not support this conclusion. Thus, further work is needed to elucidate this topic.

Finally, in pathological conditions, the opening of mPTP contributes to the release of mitochondrial Ca^2+^ into the cytosol.

### 2.2. Mitochondrial K^+^ Signalling

**Figure 2:**
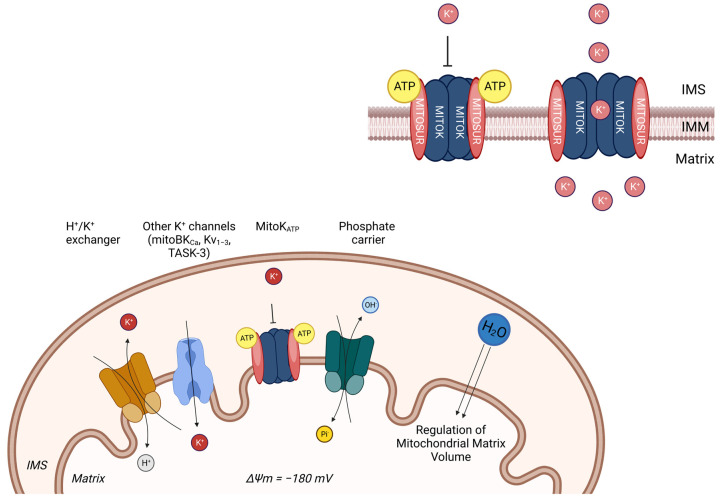
In the IMM, there are also several K^+^ channels: the ATP-regulated K^+^ channels (mitoK_ATP_), the large-conductance Ca^2+^-activated K^+^ channels (mitoBK_Ca_), voltage-dependent K^+^ channels (mitoKv_1.3_), and twin-pore TASK-3 K^+^ channels. On the contrary, excessive [K^+^] in the mitochondrial matrix is extruded by the K^+^/H^+^ antiporter, which uses the energy stored in the proton gradient to extrude K^+^ ions. The high mitochondrial electrochemical membrane potential (ΔΨ_m_ = −180 mV) represents the driving force for K^+^ influx across the IMM. Despite the negative membrane potential, in resting conditions, the [K^+^] inside and outside mitochondria are comparable. This is due to the low activity of K^+^ channels coupled to the high activity of a K^+^/H^+^ exchanger. However, when K^+^ influx is activated, it is immediately followed by anion uptake and entry of osmotically obligated water inside the matrix. On the right, the mitoKATP channel is a K^+^ channel in the IMM. It is a tetramer formed by four structural subunits (mitoKATP) inserted in the IMM and by four regulatory subunits (MitoSUR) with ATP-binding domains. A decrease in intracellular ATP levels triggers its opening, ensuring potassium accumulation in the mitochondrial matrix, regulating mitochondrial matrix volume.

Potassium is an ion responsible for the maintenance of mitochondrial volume as well as bioenergetics, which overall prevent excess matrix swelling under normal physiology [54]. The high mitochondrial electrochemical membrane potential represents the driving force for the K^+^ influx across the IMM. Despite the negative membrane potential, in resting conditions, the [K^+^] inside and outside the mitochondria are analogous. This is due to the low activity of the K^+^ channel coupled to the high activity of K^+^/H^+^ exchangers. However, when K^+^ influx is activated, it is immediately followed by anion uptake and the entry of osmotically obligated water inside the matrix. As a consequence, K^+^ homeostasis is thought to play a major role in the regulation of matrix volume, triggering cardio and neuroprotection [55] (Figure 2).

As K^+^ is the major monovalent cation in the cytosol (~150 mM), the electrogenic transport of this ion inside the mitochondrial matrix is strictly channel-dependent and highly regulated. Up to now, different mitochondrial K^+^ channels have been identified in the IMM such as mitochondrial large-conductance calcium-activated potassium (mitoBKCa) channels, mitochondrial intermediate-conductance calcium-activated potassium (mitoIKCa) channels, mitochondrial small-conductance calcium-activated potassium (mitoSKCa) channels, mitochondrial sodium-activated potassium (mitoSlo2) channels, mitochondrial voltage-regulated potassium (mitoKv) channels, and mitochondrial two-pore domain potassium (mitoTASK) channels [56,57,58,59].

This function of maintaining the mitochondrial volume is majorly carried out by ATP-dependent channels. ATP-sensitive potassium (K_ATP_) channels act as cellular metabolism sensors and are located on the plasma membrane and intracellular membranes as in the case of mitochondria as mitoK_ATP_ [59]. In the mitochondria, the negative mitochondrial membrane potential allows for the mitoK_ATP_-mediated electrophoretic uptake of K^+^ ions, which, in turn, could be inhibited by the physiological levels of ATP. MitoK_ATP_ channels are composed of pore-forming subunit MITOK and ATP-binding subunit MITOSUR (Figure 1) [60]. MITOK, encoded by the gene *CCDC51*, impairs mitochondrial homeostasis when overexpressed. Evidence also proves that MITOK is responsible for pharmacological preconditioning with diazoxide in mitoK-knockout mice; they also reported that diazoxide increases the ROS in the wild type but not in knockout conditions. Moreover, mitoK_ATP_ plays a pivotal role in the cellular signalling pathway for protection against ischemia.

However, to ameliorate targetable mitoK^+^ channels, researchers must consider some important aspects: (i) the specificity of mitoK^+^ channels according to tissue type, (ii) The relative expression of the channel in different tissue types, iii. the composition of the channel, and iv. its effect on mitochondrial bioenergetics in upregulated and downregulated conditions. For instance, cardiomyocytes express fewer mitoK_ATP_ channels compared to those in the brain, and similarly, keratinocytes express only a single type of potassium channel. Moreover, unlike mitochondrial calcium channels, mitoK^+^ channels are tissue-specific, and expression differs from tissue to tissue. So, to have a potential therapeutic targetable channel, one must ascertain the abundance of the channel in the specific tissue type.

## 3. Mitochondrial Control of Inflammation

### 3.1. Innate Immunity Receptors

The immune system is one of the most critical multiplexes of combined mechanisms, organs, tissues, cells, and proteins collaborating to prevent infections. It has been established that immunity is divided into innate, acquired, and passive immunity. This review focuses on innate immunity, which represents the first line of defence against pathogens. It is usually unspecific, provides immediate response, and is antigen-independent.

Innate immunity relies on a large family of receptors called germline-encoded PRRs, which recognize the presence of microbial components called pathogen-associated molecular patterns (PAMPs) or damage-associated molecular patterns (DAMPs) [4]. PAMPs are a microorganism’s components that target the host cell to discriminate “self” from “non-self” and activate innate immunity. Major PAMPs are microbial nucleic acids (e.g., ssRNA, dsRNA), lipoproteins, surface glycoproteins, and membrane components (e.g., lipopolysaccharides, lipoteichoic acid). Conversely, DAMPs are cell-derived molecules produced by the organism in response to tissue damage, trauma, or ischemia. The most common DAMPs are heat-shock proteins (HSPs), uric acid, heparin, ATP, and chromatin-associated proteins (HMGB1) [61]. Mitochondria seem to have a fundamental role in the control of inflammation since they contain several DAMPs, whose secretion is a common activator of inflammation [3]. Moreover, they provide a scaffold for some PRRs’ activation [62]. Finally, mitochondria can also sense danger signals and promote inflammation by activating and controlling the innate immune system [63].

PRRs can be classified based on their protein domain homology into five classes: Toll-like receptors (TLRs), C-type lectin receptors (CLRs), RIG-I-like receptors (RLRs), absent in melanoma-2 (AIM2)-like receptors (ALRs), and nucleotide-binding oligomerization domain-like receptors (NLRs) [64]. The PAMPs/DAMPs bind PRRs and trigger inflammatory pathways to eliminate microbial infections. In detail, the activation of downstream signalling pathways can recruit and release inflammatory cytokines, chemokines, hormones, and growth factors to induce the inflammatory response and subsequently initiate the acquired response [65].

PRRs recall and activate protein kinases, adaptor proteins, and transcription factors; their signals crosstalk and can converge into several common signalling pathways [64], such as the Nf-KB pathway, the mitogen-activated protein kinase (MAPK) pathway, the TBK1-IRF3 pathway, and the inflammasome signalling pathway [64]. In this chapter, we will briefly describe TLRs and NLRs since they are mainly involved in inflammatory responses.

TLRs were the earliest PRRs studied that are strongly involved in the maintenance of inflammatory responses [66]. They are expressed in all the innate immune cells, mediating the recognition of PAMPs and DAMPs in the extracellular and intracellular environment. Upon their activation, they recruit various adaptors, including the Nf-KB signalling pathway, leading to the transcription of pro-inflammatory cytokines [63].

NLRs are innate cytosolic sensors mainly involved with large macromolecular complexes, called inflammasomes, leading to the processing and activation of pro-inflammatory cytokines IL-1ß and IL-18 [67]. Importantly, a high incidence of genetic mutations in NLRs is associated with the development of chronic inflammatory diseases and autoimmune disorders [64].

Amongst all the NLRs, NLRP3, NLRP1, and NLRC4 are functionally related to their ability to form cytosolic multiprotein complexes called inflammasomes [68,69]. The term “inflammasome” was first described by Martinon and co-workers in 2002 as a process that involves the assembly of cytosolic proteins activated by the immune system, leading to the proteolytic activation of pro-inflammatory caspases [68]. Specifically, the assembly and activation of inflammasome trigger the cleavage of pro-caspase-1 into active caspase-1, which performs proteolytic cleavage of pro-IL-1β and pro-IL-18 into biologically active IL-1β and IL-18 [70].

Mature IL-1β and IL-18 are gatekeeper cytokines that mediate potent pro-inflammatory responses. In particular, IL-1β induces the expression of genes involved in fever control, pain, vasodilation, and hypotension, while IL-18 is responsible for IFN production [71]. In addition, activated caspase-1 cleaves Gasdermin-D (GSDMD), which forms pores of 18 nanometres in diameter in the plasma membrane, allowing the secretion of IL-1β, IL-18, and alarmins which, in turn, leads to a pro-inflammatory pattern of cell death called pyroptosis [72]. The GSDMD protein is formed by two distinct domains, the inhibitory C-terminal domain (GSDMD-CT) and the active N-terminal one (GSDMD-NT). Caspase-1 is responsible for the proteolytic cleavage of GSDMD, releasing the biologically active GSDMD-NT that, in turn, oligomerizes to form the pore in the plasma membrane [73]. Inflammasomes formed by NLR sensors or AIM2 and pyrin are called “canonical”, and they recruit pro-caspase-1; while the assembly of the so-called “non-canonical inflammasomes” involves human pro-caspase-4/5 and murine pro-caspase-11.

NLRP1 was the first inflammasome discovered in 2001 by Tschopp’s laboratory [68]. It is turned on in response to pathogenic enzymes (e.g., B. anthracis) [74].

The NLRC4 inflammasome was firstly described in 2004 [75]. It contains the NLRC4 sensor which interacts directly with NLR-family apoptosis inhibitor proteins (NAIPs) in response to PAMPs coming from intracellular bacteria (e.g., flagellin) [65]. It seems to be abundant in mucosal barriers protecting from invading bacteria [76].

Finally, “non-canonical” inflammasomes involve caspases-4 and -5 in humans and caspase-11 in mice. These are bi-functional molecules since they are both LPS sensors and inflammasome effectors [77].

Among NLRs, the NLRP3 inflammasome is the most studied and described in the literature, even if its activation mechanism has not been completely clarified yet [78].

### 3.2. NLRP3 Inflammasome

The NLRP3 inflammasome is crucial to defend the organism against pathogen infections associated with bacteria, fungi, and viruses; however, its overactivation can be linked to the pathogenesis of many autoinflammatory diseases, including cryopyrin-associated periodic syndrome (CAPS), Alzheimer’s diseases, gout, diabetes, and atherosclerosis.

The NLRP3 inflammasome is formed by a sensor protein, which is NLRP3 itself; an adaptor ASC or PYCARD; and an effector caspase-1. The NLRP3 sensor contains an N-terminal pyrin domain (PYD), a C-terminal domain enriched in leucine (LRR domain), and a central NACHT domain with ATPase activity that is responsible for NLRP3 self-association and function [79]. The adaptor protein ASC interacts with the N-terminal PYD protein and C-terminal caspase recruitment domain (CARD) of the caspase [80]. In detail, caspase-1 is composed of a CARD N-terminal domain, a central catalytic domain (p20), and a C-terminal small catalytic subunit (p10) [81]. Upon stimulation, the NLRP3 inflammasome is assembled and activated. Firstly, NLRP3 sensor proteins oligomerize through homotypic interactions between NATCH domains. Then, they recruit adaptor proteins through PYD–PYD interactions. Next, assembled ASCs recruit effect proteins through CARD–CARD interactions and induce self-cleavage of pro-caspase-1 between the p20 and p10 domains [82]. Finally, the cleaved and activated caspase-1 performs proteolytic cleavage of pro-IL-1β and pro-IL-18 into biologically active IL-1β and IL18.

Recent studies revealed that the assembly of the NLRP3 inflammasome requires the presence of a serine-threonine kinase (NEK7) involved in mitotic cycle progression. In detail, NEK7 interacts with the central LRR domain, exerting a scaffold function and promoting inflammasome assembly during cell interphase [83]. The absence of NEK7 prevents caspase-1 activation and IL-1β release in vitro and in vivo [84].

**Figure 3:**
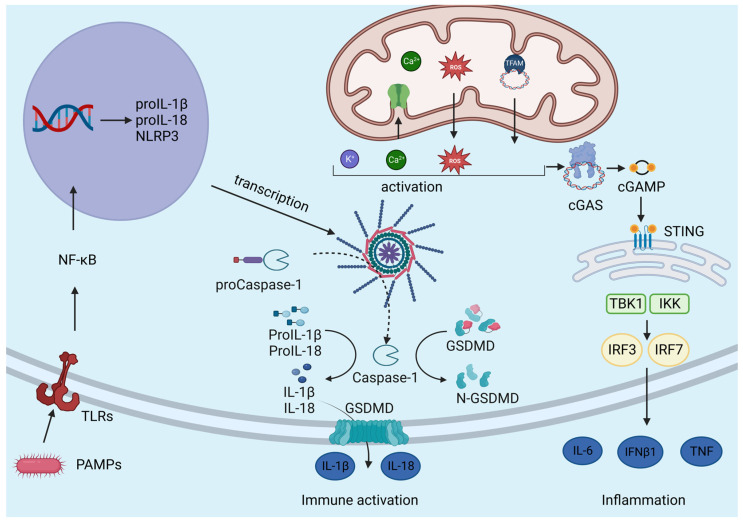
NLRP3 is a cytosolic protein complex. It triggers the cleavage and activation of caspase-1, which is responsible for the cut and activation of pro-inflammatory cytokines (IL-1β and IL-18). Moreover, it causes the cut and the oligomerization of GSDRM monomers at the plasma membrane level, forming a pore to release IL-1β and IL-18, starting the pro-inflammatory signalling cascade and the pyroptotic cell death. The NLRP3 inflammasome induction requires the coexistence of two signals. The “first signal or priming” (on the left) is carried out by PAMPs, leading to the transcriptional upregulation of NLRP3 to induce the inflammasome component’s transcription. The “second signal” (in the centre) is activated by multiple upstream signalling events (e.g., ion fluxes, lysosomal disruption, and mtDNA release upon mitochondrial damage), and it is responsible for NLRP3 inflammasome activation. mtDNA release in the cytosol can also trigger the activation of cGAS-STING pathway (on the right). cGAS binds DNA in the cytosol and synthetizes GMP-AMP (cGAMP) from ATP and GTP. cGAMP binds STING located in ER membrane. Activated STING then recruits and activates TBK and IRF3, which leads to the production of type I interferons and other pro-inflammatory cytokines.

The inflammasome activation must be tightly regulated to ensure a specific and localized immune response to pathogen infections. NLRP3 inflammasome activation consists of a two-step mechanism that requires the combination of a plethora of signals [85] (Figure 3). The first signal, also called priming, promotes the transcription of inflammasome components, while the second signal induces their post-translational modifications (PTMs), promoting their assembly and the activation of the NLRP3 platform [86].

The first signal is provided by microbial molecules, PAMPs and DAMPs, which bind TLRs, NLRs, or cytokine receptors in the plasma membrane and activate the inflammatory signalling cascade, promoted by MyD88 and TRIF adaptors and leading to the activation of NF-kB signalling [87]. Activated NF-kB moves into the cell nucleus where it binds the promoter of several inflammasome component genes such as NLRP3, pro-IL-1β, and pro-IL-18 [78]. However, priming does not appear to affect ASC and pro-caspase-1 transcription [87]. On the contrary, the second signal includes a variety of upstream signals that are not mutually exclusive, such as K^+^ efflux, Ca^2+^ flux, lysosomal disruption, and mitochondrial dysfunction [88,89]. Although there is no consensus model for NLRP3 activation, it is generally accepted that mitochondria play a crucial role by participating in ion homeostasis and releasing mtDNA and ROS [87]. To sum up, mitochondrial damage seems to be the most widely studied activating stimulus for the NLRP3 inflammasome since it connects diverse inflammatory, metabolic, and malignant diseases.

As for K^+^ efflux, a decreased intracellular K^+^ concentration [K^+^] has been considered typical for inflammasome activation. Indeed, it was demonstrated that the cytosolic depletion of K^+^, induced by nigericin, an ionophore that allows intracellular K^+^ efflux across the membrane, mediates IL-1β maturation upon inflammasome activation [7].

Also, Ca^2+^ seems to be involved in NLRP3 inflammasome activation, even if the mechanism by which increased [Ca^2+^]_cyt_ can induce NLRP3 inflammation is not yet clear. Ca^2+^ enters into the cytosol through the opening of store-operated Ca^2+^ entry (SOCE) located in the plasma membrane, which is often coupled with the mobilization of Ca^2+^ in the ER/SR through InsP_3_R and through the ryanodine receptors (RyR) in the SR [90,91]. As a consequence, increased [Ca^2+^]_cyt_ lead to Ca^2+^ accumulation in the mitochondrial matrix through the MCU [15,16]. However, excessive mitochondrial Ca^2+^ uptake leads to mitochondrial dysfunction, in particular, causing ROS and mtDNA release, which are responsible for NLRP3 inflammasome activation [71]. Moreover, dysfunctional mitochondria release mtDNA and drive inflammatory responses upon their accumulation in the cytosol, underlying the role of mitochondria in inflammatory responses [3]. In detail, mtDNA and mtROS are released under stress conditions. On the one hand, mtDNA interacts with both NLRP3 and AIM2 [92]; on the other hand, mtROS, generated from mitochondrial dysfunctions, are required to activate the NLRP3 inflammasome in the presence of LPS and ATP [5]. It has been demonstrated that chemical inhibitors of ROS production suppress NLRP3 inflammasome activation [92]; moreover, ROS seem to lead to inflammasome activation through an AMPK-autophagy-ROS signalling pathway induced by fatty acid [93]. On the contrary, several studies have described the role exerted by ROS in the priming phase of inflammasome activation, demonstrating that ROS inhibitors can block NLRP3 expression in the first step [87]. In conclusion, additional studies are needed to clarify the role of ROS in inflammasome activation.

### 3.3. Role of Mitochondrial Ca^2+^ Uptake in Inflammation

Until now, no consensus model for NLRP3 inflammasome activation exists. However, emerging evidence underlines the role exerted by mitochondrial Ca^2+^ flux in controlling the inflammatory response. In particular, many studies have demonstrated that the inhibition of mitochondrial Ca^2+^ uptake attenuates the inflammatory response. In detail, Gu and co-workers showed that macrophages from MCU^+/−^ mice were protected from pulmonary fibrosis [6]. Furthermore, reduced IL-13 levels were registered in MCU^−/−^ tracheal epithelial cells [94].

In addition, it has been demonstrated that mitochondrial Ca^2+^ accumulation regulates NLRP3 activation in epithelial cells of cystic fibrosis (CF) inflammatory lung disease [95]. In this regard, it has been demonstrated that mitochondrial Ca^2+^ uptake integrates pro-inflammatory signals initiated by flagellin to activate the NLRP3 inflammasome and develop an inflammatory response. To corroborate this hypothesis, CF bronchial cells treated with the MCU inhibitor KB-R7943 showed an attenuated inflammatory reaction in response to *P. aeruginosa* infection [96].

In addition, Tedesco and co-workers examined the role of mitochondrial Ca^2+^ uptake in macrophage differentiation during the phagocytosis process [97]. It was demonstrated that mitochondrial Ca^2+^ uptake is required for macrophage polarization. In particular, macrophage polarization through the pro-inflammatory M1 phenotype is promoted by extracellular Ca^2+^ influx; on the contrary, low cytosolic Ca^2+^ levels promote anti-inflammatory M2 polarization, providing higher phagocytic activity. In summary, the study of mitochondrial Ca^2+^ homeostasis in macrophages during the phagocytosis process can become a new tool for investigating the development of inflammatory responses.

Importantly, Feno and colleagues showed that MCUb^−/−^ macrophages cannot acquire the anti-inflammatory profile during skeletal muscle regeneration [98]. MCUb^−/−^ macrophages show an upregulation of pro-inflammatory cytokines, indicating that increased mitochondrial Ca^2+^ accumulation upregulates the inflammatory response.

Finally, a myeloid MCU^−/−^ mouse model (MCU^Δmye^) was developed [99]. In this study, it was demonstrated that MCU is required for phagocytosis-triggered activation of the NLRP3 inflammasome by counteracting the Ca^2+^-dependent recruitment of the endosomal sorting complex (ESCRT complex) [100,101] that mediates phagolysosome membrane repair. Overall, they concluded that importantly, mitochondrial Ca^2+^ accumulation is not a general mediator of the NLRP3 inflammasome in a phagocytic-independent pathway, since no changes in NLRP3 inflammasome activation in response to nigericin or ATP stimulation were registered.

These data confirm the crucial role exerted by mitochondrial Ca^2+^ accumulation in the control of the inflammatory response. Nevertheless, the connection between mitochondrial Ca^2+^ uptake and the first signal (priming) is not yet completely clarified.

Also, Na^+^ influx and Cl^−^ efflux are two additional events involved in NLRP3 inflammasome activation. In particular, low extracellular Cl^−^ levels seem to promote inflammasome activation. Moreover, Cl^−^ efflux is a downstream signal of both K^+^ efflux and mitochondrial dysfunctions, confirming its role in NLRP3 inflammasome activation [102].

Furthermore, lysosomal damage also seems to be involved in NLRP3 activation. In detail, phagocytosis of monosodium urate (MSU), alum, silica, asbestos, amyloid-cholesterol crystals, and Ca^2+^ crystals causes lysosomal rupture and a release of particulates in the cytoplasm, which appears to be critical for NLRP3 inflammasome activation. However, the mechanisms linking lysosomal disruption to NLRP3 inflammasome induction remain unclear. It was recently proposed that MSU crystals can activate the nucleotide-binding oligomerization domain-like receptor pyrin domain of the NLRP3 inflammasome [103]. In addition, it was also demonstrated that the acidification by MSU causes a massive Na^+^ release and water influx, resulting in decreased intracellular [K^+^] that, in turn, promotes the activation of NLRP3 [104]. All these events involved in the maintenance of the second signal are caused by cellular stress conditions, such as mitochondrial dysfunctions, lysosomal damage, and the production of ROS, and they are strongly connected to ion fluxes, in particular, K^+^ efflux and Ca^2+^ influx. Nevertheless, additional experiments are necessary to uncover the mechanisms and molecular pathways able to induce the activation of the NLRP3 inflammasome.

### 3.4. Role of Mitochondrial K^+^ Fluxes in Inflammation

Emerging research evidence suggests that K^+^ signalling in mitochondria may play a role in regulating inflammatory responses. Mitochondria have been recognized as signalling organelles involved in various cellular processes, including immune responses and inflammation. Here is an overview of the connection between mitochondrial K^+^ signalling and inflammation.

Dysregulated K^+^ efflux can lead to membrane depolarization and subsequent activation of the NLRP3 inflammasome. This inflammasome activation results in the release of pro-inflammatory cytokines, such as IL-1β and IL-18, promoting inflammation [102].

Since mitochondria are a major source of ROS, which can modulate inflammatory responses, it has been demonstrated that dysregulated K^+^ homeostasis can affect mitochondrial ROS production and redox signalling pathways, contributing to the activation of inflammatory signalling cascades. Mitochondrial K^+^ channels have been implicated in the regulation of cell death processes as well such as apoptosis and pyroptosis. Inflammatory cell death pathways can be triggered by K^+^ efflux from mitochondria, leading to the release of pro-inflammatory molecules and the activation of immune responses [105,106]. It is important to note that the precise mechanisms and signalling pathways connecting mitochondrial K^+^ fluxes to inflammation are still an active area of research. Understanding these processes may potentially uncover new therapeutic targets for the treatment of inflammatory diseases. Nevertheless, a plethora of experimental approaches have been employed to investigate the role of mitochondrial K^+^ channels in inflammation.

Pharmacological agents have been utilized to modulate the activity of mitoK^+^ channels and assess their impact on inflammation. For example, opening mitoK^+^ channels using pharmacological activators such as diazoxide or nicorandil has been shown to reduce inflammation in various experimental models [105,107,108]. Conversely, blocking these channels with inhibitors like 5-hydroxydecanoate (5-HD) can lead to increased inflammation [109]. It has to be taken into account that these pharmacological agents are not specific for mitochondrial K^+^ channels since they also work for plasma membrane K^+^ channels; thus, further work is needed to clarify this field of research.

Various animal models of inflammatory diseases have been employed to investigate the role of mitoK^+^ channels. These models include conditions like sepsis, ischemia-reperfusion injury, and neuroinflammation. By assessing the effects of mitoK^+^ channel modulation on disease severity, immune cell activation, cytokine production, and tissue damage, researchers can gain a better understanding of their involvement in inflammatory processes.

These experimental approaches, along with others, have deciphered the potential links between mitoK^+^ channels and inflammation. However, it is important to note that the field is still evolving, and further studies are needed to confirm and expand upon these findings.

### 3.5. mtDAMP

mtDAMPs, which stands for mitochondrial damage-associated molecular patterns, are molecules released by damaged or dysfunctional mitochondria that can activate the immune system and trigger inflammatory responses. mtDAMPs are recognized by specific receptors on immune cells, leading to the activation of signalling pathways that promote inflammation. Some examples of mtDAMPs include mtDNA, mitochondrial RNA (mtRNA), ATP, TFAM, and mitochondrial proteins [110,111]. When mitochondria become damaged or stressed, these molecules can be released into the cytoplasm or extracellular space. Once released, mtDAMPs can be recognized by the pattern-recognition receptors (PRRs) present on immune cells, such as Toll-like receptors (TLRs). Activation of TLRs by mtDAMPs initiates intracellular signalling cascades that trigger the production and release of pro-inflammatory cytokines, chemokines, and other inflammatory mediators [110,111]. This inflammatory response is an important defence mechanism to eliminate damaged mitochondria and maintain cellular homeostasis.

Notably, it has been reported that the mtDAMP succinate plays a pivotal role in metabolic syndromes such as diabetic ketoacidosis and in how it is upregulated in patients with PTEN, SDHB, and SDHD mutations in Cowden syndrome [112,113]. Moreover, elevated levels of mtDNA have been observed in patients with urological metastatic cancer [114]. Also, elevated levels of mtDNA in plasma usually correspond to decreased survival and serve as potential biomarkers for prognosis [115,116].

Similarly, TFAM, a member of the HMG box family, has also been shown to be involved in elucidating immune responses, acting as a DAMP under various stress conditions.

Nevertheless, the primary source of energy, ATP, also acts as a mtDAMP and has been extensively studied in various immune cells, epithelial cells, RBCs, and neurons. It is immensely intriguing to understand how elevated levels of this molecule contribute to the pathogenicity of any disease via activating its specific receptors, P2XR or P2YR [116,117]. ATP is also involved in releasing IL-1β and IL18, amongst other cytokines in immune cells like macrophages and monocytes, via activating the NLRP3 inflammasome [118].

### 3.6. cGAS-STING Pathway

The cGAS-STING pathway is a critical cellular signalling pathway involved in innate immune responses. It is responsible for detecting cytosolic DNA, which can be derived from viral infections, intracellular bacteria, or other sources of DNA damage [119]. The pathway starts with the enzyme cGAS, which stands for cyclic GMP-AMP synthase. cGAS recognizes and binds to double-stranded DNA in the cytoplasm. Upon binding to DNA, cGAS catalyses the synthesis of a molecule called cyclic GMP-AMP (cGAMP) from ATP and GTP. cGAMP is a cyclic dinucleotide and functions as a second messenger, binding to a protein called the Stimulator of Interferon Genes (STING) located on the ER membrane [119]. Upon the binding of cGAMP to STING, a conformational change occurs in STING, leading to its activation [119,120]. Then, the activated STING recruits a series of downstream signalling molecules, including TBK1 (TANK-binding kinase 1) and IRF3 (Interferon Regulatory Factor 3). The activation of TBK1 and IRF3 leads to the production of type I interferons and other pro-inflammatory cytokines essential for the immune response against viral infections and other pathogens. The cGAS-STING pathway can also induce the expression of other genes involved in antiviral defence and immune regulation.

When mtDNA is exposed to the cytoplasm, it can activate various pathways, including the cGAS pathway, and initiate an immune response. Several studies have indicated that mitochondrial Ca^2+^ plays a crucial role in modulating the cGAS pathway [121]. Changes in intracellular Ca^2+^ levels have been linked to mitochondrial stress and damage, leading to the release of mtDNA into the cytosol [122]. Elevated cytosolic Ca^2+^ levels can activate the cGAS enzyme and stimulate the production of cyclic GMP-AMP (cGAMP). A study conducted on primary mouse bone marrow-derived macrophages (BMDMs) and human embryonic kidney (HEK293T) demonstrated that mitochondrial Ca^2+^ uptake promoted cGAS activation and subsequent interferon production in response to cytosolic DNA stimulation. In detail, increased mitochondrial Ca^2+^ uptake, using the Ca^2+^ ionophore A23187, enhanced cGAS activation and the expression of interferon-stimulated genes [123]. Conversely, the inhibition of mitochondrial Ca^2+^ accumulation with the Ca^+2^ chelator BAPTA-AM reduced cGAS-mediated interferon production. Studies conducted on BMDMs extracted from C57BL/6 mice also revealed an upregulated uptake of Ca^2+^ by mitochondria potentiating mPTP (mitochondrial permeability transition pore) opening and oligomerization of the VDAC channel which resides on the OMM [121]. This resulted in the leakage and release of oxidized mtDNA from the inner mitochondrial membrane (IMM) in the cytoplasm, eventually leading to the activation of the NLRP3 inflammasome. Interestingly, this process takes place in the absence of LPS-mediated priming, and the effects increase to many folds in the presence of LPS priming.

Mitochondrial nucleases such as Fen1 (flap structure-specific endonucleases) and Mgme1 (mitochondrial genome maintenance exonuclease-1) are also found to be responsible for the release of mtDNA in the cytosol and the activation of NLRP3 inflammasome complexes [124]. Moreover, as a sequence of events, it also leads to the activation of the cGAS-STING pathway in the cytosol [125]. Ox-mtDNA has been frequently linked to IL-1β activation and release, circulating cell-free mtDNA release, and the phosphorylation of STING [126].

Inflammatory and metabolic pathophysiological conditions, such as heart failure, atherosclerosis, NASH, Alzheimer’s disease, and rheumatoid arthritis are often associated with Ox-mtDNA release in the cytosol, activation of the NLRP3 inflammasome [92] and cGAS-STING pathway; however, to date, it is not quite well-understood. Mitochondrial cations, particularly Ca^2+^ and K^+^, play crucial roles in modulating the cGAS pathway. Changes in intracellular Ca^2+^ levels have been linked to mitochondrial stress and damage, leading to the release of mtDNA into the cytosol. Elevated cytosolic Ca^2+^ levels can activate the cGAS enzyme and stimulate the production of cyclic GMP-AMP (cGAMP) [121].

Collectively, these studies provide experimental evidence highlighting the regulatory role of mitochondrial Ca^+2^ in the cGAS pathway. They demonstrate that changes in mitochondrial Ca^+2^ levels can influence cGAS activation, indicating a direct link between mitochondrial homeostasis and the innate immune response.

## 4. Diseases Associated with NLRP3 Inflammasome Dysfunction

One of the critical features of the NLRP3 inflammasome is its ability to sense the presence of PAMPs/DAMPs and activate the inflammatory signalling cascade. However, excessive and persistent NLRP3 inflammasome activation is linked to various chronic diseases [127]. Here, we discuss some examples of chronic inflammatory diseases caused by persistent activation of the NLRP3 inflammasome.

**Lung inflammatory diseases**. The excessive and chronic activation of the NLRP3 inflammasome can lead to bacterial infections, asbestosis, silicosis, severe asthma, COPD, and cystic fibrosis [128]. For example, Kim and co-workers found increased NLRP3 and IL-1β responses in steroid-resistant asthma patients [129]. Moreover, neutrophilic asthmatic patients showed a significant increase in the gene expression of NLRP3, caspase-1, caspase-4, caspase-5, and IL-1β [130].

In 2020, Rimessi’s group demonstrated that the inflammatory response in cystic fibrosis (CF) is related to overactivation of the NLRP3 inflammasome. In detail, CF in bronchial cells is supported by *P. aeuriginosa* perturbation, which alters intracellular Ca^2+^ homeostasis, leading to the overactivation of the NLRP3 inflammasome [96]. In this regard, it has been demonstrated that mitochondrial Ca^2+^ uptake integrates pro-inflammatory signals initiated by flagellin to activate the NLRP3 inflammasome and develop an inflammatory response. To corroborate this hypothesis, CF bronchial cells treated with the MCU inhibitor KB-R7943 showed an attenuated inflammatory reaction in response to *P. aeruginosa* infection [96].

Finally, chronic obstructive pulmonary disease (COPD) is a serious pulmonary disease characterized by airway obstruction and inflammation. Cigarette smoke seems to be a higher risk factor for COPD patients, recruiting a variety of pro-inflammatory cytokines and cells [131]. In particular, it has been demonstrated that cigarette smoke extract induces pyroptosis in human bronchial epithelial cells through the NLRP3/caspase-1 signalling pathway. So, the NLRP3 inflammasome seems to have an essential role in the progression of COPD pathogenesis [132].

**Bowel inflammasome diseases.** Bowel inflammatory diseases (IBD), like Crohn’s disease and ulcerative colitis, are characterized by an intense and chronic activation of the NLRP3 inflammasome in the gastrointestinal tract [133,134,135]. The severity of IBD is clinically associated with IL-1β and IL-18 levels, both of which promote the differentiation and amplification of pro-inflammatory T cells [136]. Interestingly, intestinal microbiota seems to be related to NLRP3 inflammasome activation in IBD. It has been demonstrated that faecal bacteria from Chron’s patients are more effective at upregulating NOD2, NLRP3, and TLR expression than those from healthy controls [6].

The role of the NLRP3 inflammasome in IBD pathogenesis has been investigated both in animal and human models, even if the exact role of the NLRP3 inflammasome is still controversial since it seems to exert both pathogenic and protective effects [137]. For example, people with mutations at the level of IL-10R spontaneously develop CD, suggesting that IL-10 is fundamental to restoring the immunosuppressive activity and promoting the anti-inflammatory pathway [138]. On the contrary, it has been shown that NLRP3-, ASC-, and caspase-1-deficient mice develop more severe experimental colitis [139]. In regards to human studies, an increased IL-1β and IL-18 secretion from the colonic tissues of IBD patients is correlated to the pathogenesis of the disease by promoting chronic intestinal inflammation [140].

In addition to the established link between the NLRP3 inflammasome and IBD, it is noteworthy that mitochondrial dysfunctions contribute to the impairment of intestinal epithelial barrier function, thereby playing a significant role in the pathogenesis of IBD. Subsequently, we will present supporting data for this hypothesis. For a comprehensive review, please refer to [141].

Mice treated with dextran sodium sulfate (DSS), a chemical compound known to induce intestinal inflammation, exhibit mitochondrial dysfunction characterized by increased ROS production, decreased ATP, and mitochondrial swelling [142]. Furthermore, alterations in the mitochondrial structure, including cristae remodelling and intramitochondrial vesicle accumulation, have been observed [143]. In human studies, it has been demonstrated that reduced ATP production during intestinal inflammation is associated with decreased COX subunit levels [144]. Additionally, IBD patients display elevated levels of circulating mitochondrial DNA (mtDNA) in the plasma, and notably, this feature correlates with the severity of the disease [145]. However, conflicting findings indicate that the absence of mtDNA can trigger the expression of inflammatory cytokines, such as IL-8, in colon cells [144]. Consequently, further research is warranted to elucidate the precise role of mtDNA in the regulation of intestinal inflammation.

Patients with ulcerative colitis manifest decreased levels of mitochondrial respiratory chain complexes in the mucosa [146]. To reinforce this observation, animals exhibiting heightened ATP levels and mitochondrial respiratory chain activity demonstrate less severe manifestations of ulcerative colitis [147]. These findings collectively underscore the fundamental role of mitochondrial function in the development of IBD pathogenesis.

In conclusion, more studies are needed to determine the role of the NLRP3 inflammasome in the pathogenesis of IBD. However, its activation is crucial for initiating the inflammatory process, which results in tissue damage and IBD manifestation. Nevertheless, the protective role of the NLRP3 inflammasome during inflammation could be a compensatory mechanism to maintain intestinal homeostasis [137].

**Metabolic disorders.** The NLRP3 inflammasome has also been directly associated with the pathogenesis of metabolic diseases. In particular, the interconnection between the immune system and the metabolic system is essential to balance metabolic homeostasis [69]. The NLRP3 inflammasome contributes to many metabolic diseases such as diabetes and obesity [148,149]. For example, some danger signals related to obesity, such as palmitate, lipids, and ceramides, are involved in NLRP3 inflammasome activation. In detail, saturated fatty acids, promote mtROS accumulation through the AMPK signalling pathway, leading to inflammasome activation as well as insulin resistance [93]. Similarly, obesity-related lipotoxic ceramides activate caspase-1 in an NLRP3-dependent manner [150]. Finally, elevated homocysteine levels can act as a DAMP, providing the second signal to activate the NLRP3 inflammasome through the induction of the HIF1α transcription factor, generating adipocyte-derived lysophosphatidylcholine (lyso-PC) [151]. In conclusion, NLRP3 activation causes IL-1β release, increasing insulin resistance and reducing glucose uptake in target tissues such as liver and adipose tissue, leading to the pathogenesis of diabetes and obesity [69].

## 5. Conclusions

Although ample data support the critical roles of mitochondrial cations in inflammation and in diseases, many facts remain to be uncovered.

In particular, it is still not clear how mitochondrial cations are central in immune responses and in pathological conditions. In addition, it is evident that mitochondrial Ca^+2^ and K^+^ ions are crucial for sustaining cellular homeostasis, bioenergetics, as well as inflammatory responses.

Mitochondria as a signalling hub are also responsible for the production and release of DAMPs, which induce inflammation by activating the downstream cGAS-STING pathway. Overall, it is important to decipher the molecular mechanisms by which mitochondrial cations as well as mitochondrial molecules can induce inflammation. These findings will provide a significant advance in the identification of promising therapeutic targets.

## Data Availability

No new data were created.
